# UNDERSTANDING THE IMPACT OF BPSD ON CAREGIVER HEALTH OUTCOMES FROM THE PARADIGM OF SOCIAL INTERACTION

**DOI:** 10.1093/geroni/igae098.2366

**Published:** 2024-12-31

**Authors:** Ziying Yang, Doris Sau Fung Yu, Polly Wai Chi Li

**Affiliations:** The University of Hong Kong, Hong Kong, China (People’s Republic); The University of Hong Kong, Hong Kong, China (People’s Republic); The University of Hong Kong, Hong Kong, China (People’s Republic)

## Abstract

Behavioral and Psychological Symptoms of Dementia (BPSD) in persons with dementia (PwD) is a crucial determinant of caregiver health outcomes. With increasing evidence on factors associating with BPSD, it is imperative to develop a theoretical model to elucidate the mechanisms behind this association. This study aimed to develop and test an integrative model based on an integrative stress and coping, stress-vulnerability, and the social interactional paradigm to explain the impact of BPSD on caregiver health outcomes. A total of 200 family caregivers of PwD were recruited from three tertiary hospitals in China. Validated questionnaires were used to measure the BPSD, caregivers’ expressed emotion (EE), their attribution about patients’ BPSD and neurotic personality. Dyadic relationship strain was measured. The caregivers’ health outcomes were captured by their health-related quality of life (HRQL) and depression. By using path analysis (AMOS 26.0), our findings indicated that BPSD independently predicted a higher level of depression and poorer mental HRQL of family caregivers. The explanatory pathways highlighted the roles of caregivers’ EE and relationship strain in mediating the impact of patients’ BPSD on caregiver depression (β=0.049, p<0.001) and mental HRQL (β=-0.037, p<0.001). Caregivers of higher neuroticism was even more susceptible to depression (β=0.033, p<0.001) and poorer mental HRQL (β=-0.025, p<0.001) and their maladaptive attribution, poorer emotional regulation and relationship quality aggravate the negative response to BPSD. These findings inform interventions would provide highly specific insights to increase the caregivers’ adaption to the BPSD in a dementia caregiving context.

